# Supplementary material for: Tracking momentary fluctuations in human attention with a cognitive brain-machine interface
Source: Commun Biol. 2022 Dec 8;5:1346. doi: 10.1038/s42003-022-04231-w (PMC9732358; doi:10.1038/s42003-022-04231-w)
Supplement: Supplementary file 2 — Supplementary Information [file 42003_2022_4231_MOESM2_ESM.pdf]

Supplementary Information

Tracking momentary fluctuations in human attention with a cognitive brain-machine interface

Abhijit M. Chinchani, Siddharth Paliwal, Suhas Ganesh, Vishnu Chandrasekhar, Byron M. Yu, Devarajan Sridharan*

*Correspondence to: Devarajan Sridharan
Email: sridhar@iisc.ac.in

Supplementary Figure 1

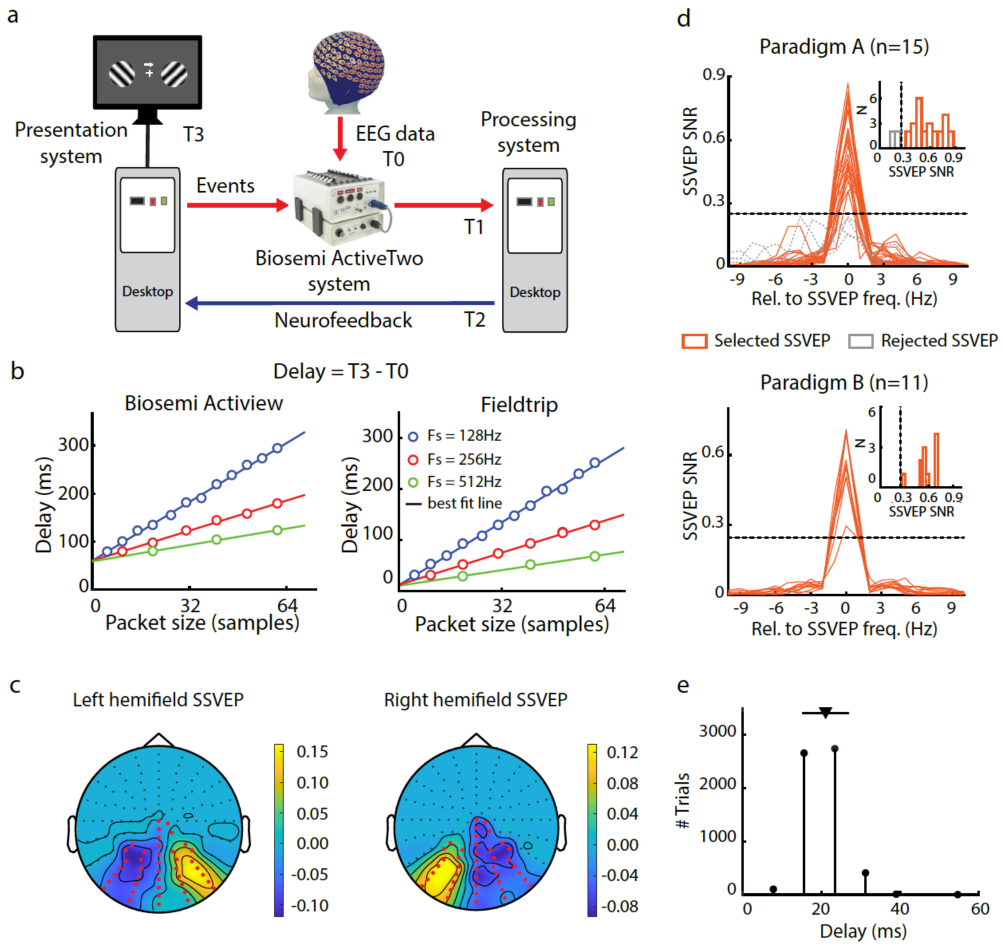

Supplementary Figure 1. Results auxiliary to Figure 1 (main text).

a. Schematic of the cBMI system (see main Methods section Closed-loop Cognitive Brain Machine Interface (cBMI) based on EEGs., for details).

b. (Left) Closed-loop delay – the time between EEG data acquisition and neurofeedback -- for different packet sizes using the Biosemi™ Actiview acquisition software. Different colors represent distinct sampling frequencies (Fs): 128 Hz (blue), 256 Hz (red), 512 Hz (green). Circles: closed-loop delays averaged across 500 iterations; lines: best-fit lines. (Right) Same as in the left panel but with the Fieldtrip acquisition software.

c. Topographic plot indicating the normalized SSVEP DSS dimension, averaged across all participants. Red dots: locations of the 41 occipital EEG electrodes used in the study. Black dots: locations of remaining (unused) electrodes.

d. Signal to Noise Ratio (SNR) for the SSVEP DSS dimensions for each subject (distinct lines) for paradigm A (top) and paradigm B (bottom) (see main Methods section EEG and behavioral data analysis., for details). Dashed black horizontal line: SNR=0.25. Solid orange lines: SSVEP dimensions used for analysis. Dashed thin gray lines: SSVEP dimensions excluded from analysis. Inset: histogram of SNR at the SSVEP frequency for each component. Orange and gray bars: included and excluded dimensions, respectively. Dashed vertical line in inset: SNR=0.25.

e. Distribution of the measured closed-loop delay in all the trials, across all subjects in paradigm A and B. The inverted triangle and the error bar on top of the histogram indicate the mean and standard deviation of the delay.

Supplementary Figure 2

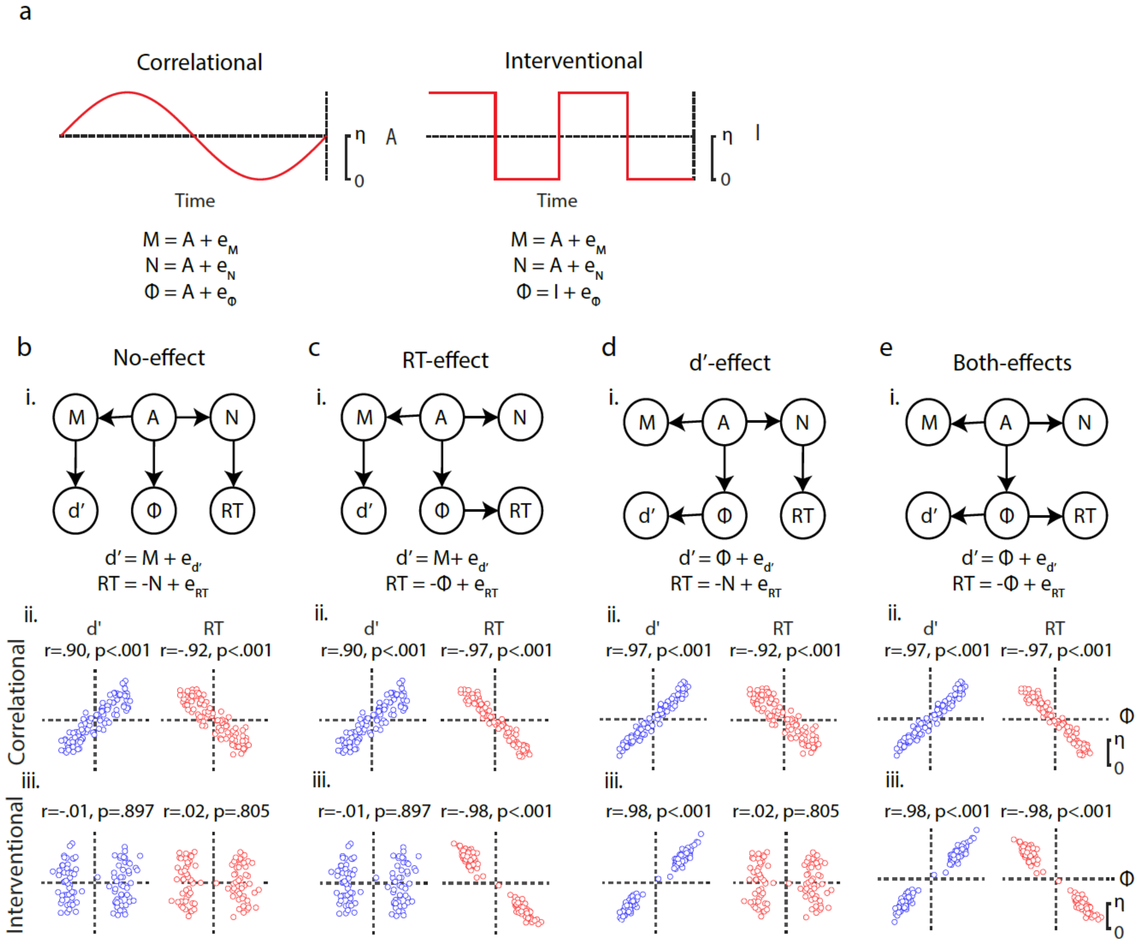

Supplementary Figure 2.

a. Time course of source signal (A(t)) (left) and intervened signal (I(t)) in (right). Equations describing the relationships between the neural processes M, N, Φ and source signal A for the Correlational approach (left) and Interventional approach (right). Here, A(t) represents the attention signal, Φ(t) is the SSVEP power (CDF normalized, see text for details), M and N are alternative neural processes distinct from Φ, d' is discrimination accuracy, and RT is reaction time. $e_X(t)$ represent additive noise, for the respective neural process (X) drawn from a unit normal distribution independently at each time instant.

b. (i) Schematic depicting the No-effect model of attention's effect on d' and RT. (ii) Scatter plot showing relationship between Φ and d' (ii, left sub-panel, blue) or between Φ and RT (ii, right sub-panel, red data) for the Correlational approach. (iii) Same as panel ii, but for the Interventional approach. In each subpanel, correlation coefficients (r) and significance values (p) are indicated above each subpanel.

c. Same as in panel b, but for the RT-effect model.

d. Same as in panel b, but for the d'-effect model.

e. Same as in panel b, but for both-effects model.

(c-e). Other conventions are the same as in panel b.

Supplementary Figure 3

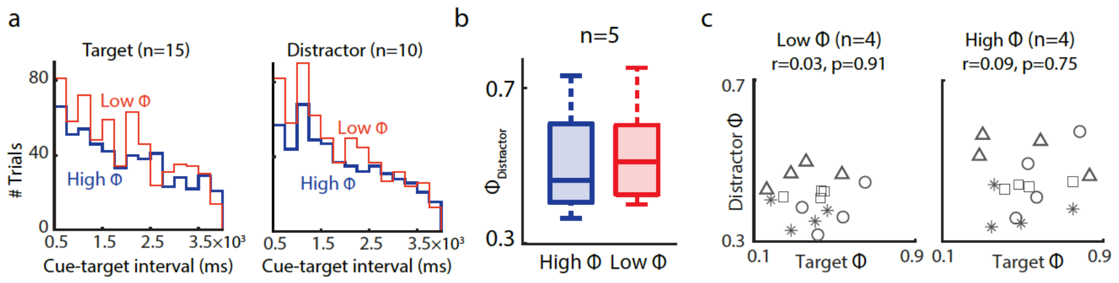

Supplementary Figure 3. Results auxiliary to Figure 2 (main text).

a. Distribution of cue-target intervals for all trials pooled across subjects, plotted separately for high-Φ (blue) and low-Φ (red) trials (Left: Target triggered, Right: Distractor triggered).
b. Φ on the distractor side for High-Φ (blue box) and Low-Φ (red box) target triggered trials. Central mark: median, edges: 25th and 75th percentiles, the whiskers: most extreme points.
c. Trial-averaged correlation between target Φ and distractor Φ for Low-Φ (left) and High-Φ (right) trials for Paradigm A. Distinct symbol represents data from different subjects and each symbol corresponds to Φ computed in a specific time window prior to grating triggering (see Methods for details).

Supplementary Figure 4

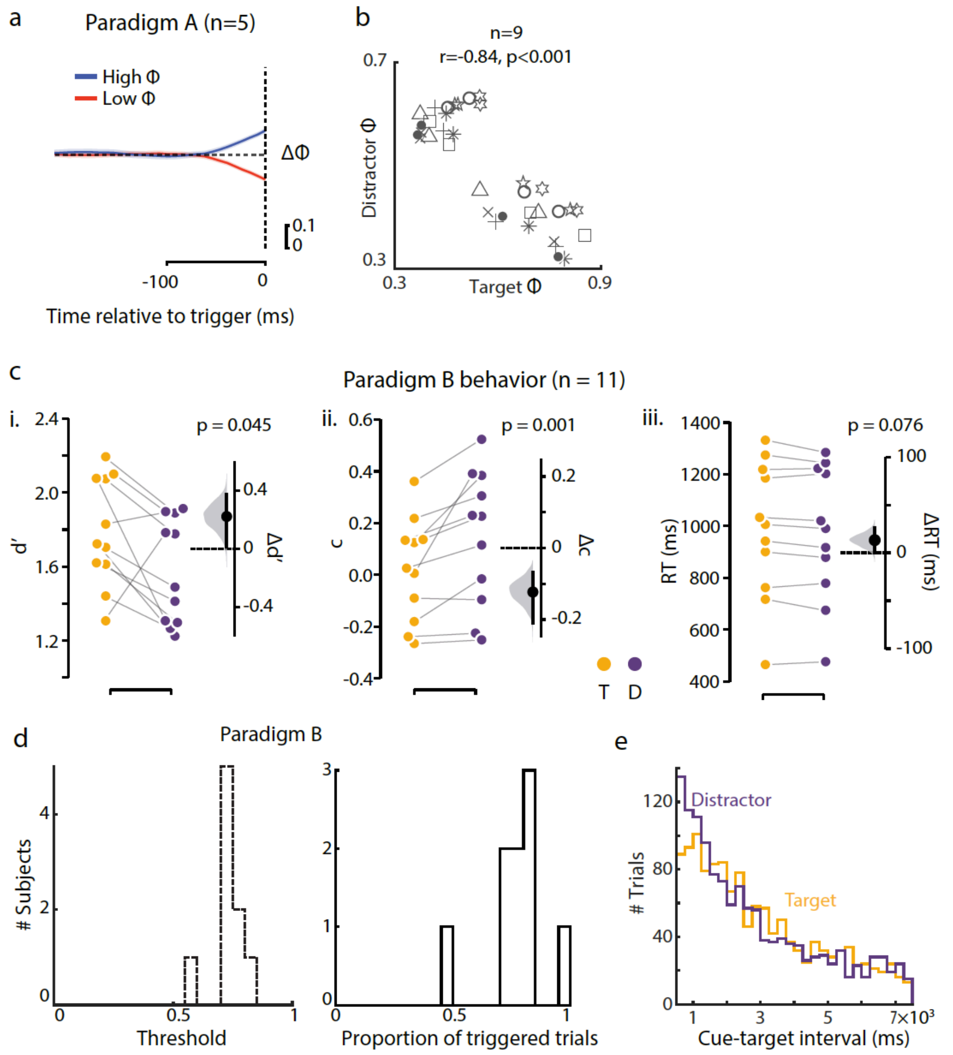

Supplementary Figure 4. Results auxiliary to Figures 3-4 (main text).

a. Mean ΔΦ traces for high-Φ (blue) and low-Φ (red) target triggered trials in Paradigm A, time-locked to the presentation of the grating stimuli (dashed vertical line). Shaded region: s.e.m.

b. Trial-averaged correlation between target Φ and distractor Φ for Paradigm B. Distinct symbol represents data from different subjects and each symbol corresponds to Φ computed in a specific time window prior to grating triggering (see Methods for details).

c. Same as in Fig. 4e (main text) but with all subjects tested in paradigm B (n=11), including the two subjects repeated from paradigm A (see Methods, Supplementary Table 1). Black dot and errorbar: Mean effect size and 95% confidence interval, respectively, for the difference between d' across target and distractor probed trials. Gray histogram: bootstrap sampling distribution for effect size. Dashed horizontal line: Datum indicating zero effect size.

d. Distribution of ΔΦ thresholds (left) and number of triggered trials (right) for paradigm B (n=9 participants).

e. Distribution of cue-probe intervals for trials, pooled across all subjects, plotted separately for target-probed (yellow) and distractor-probed (purple), in paradigm B.

Supplementary Figure 5

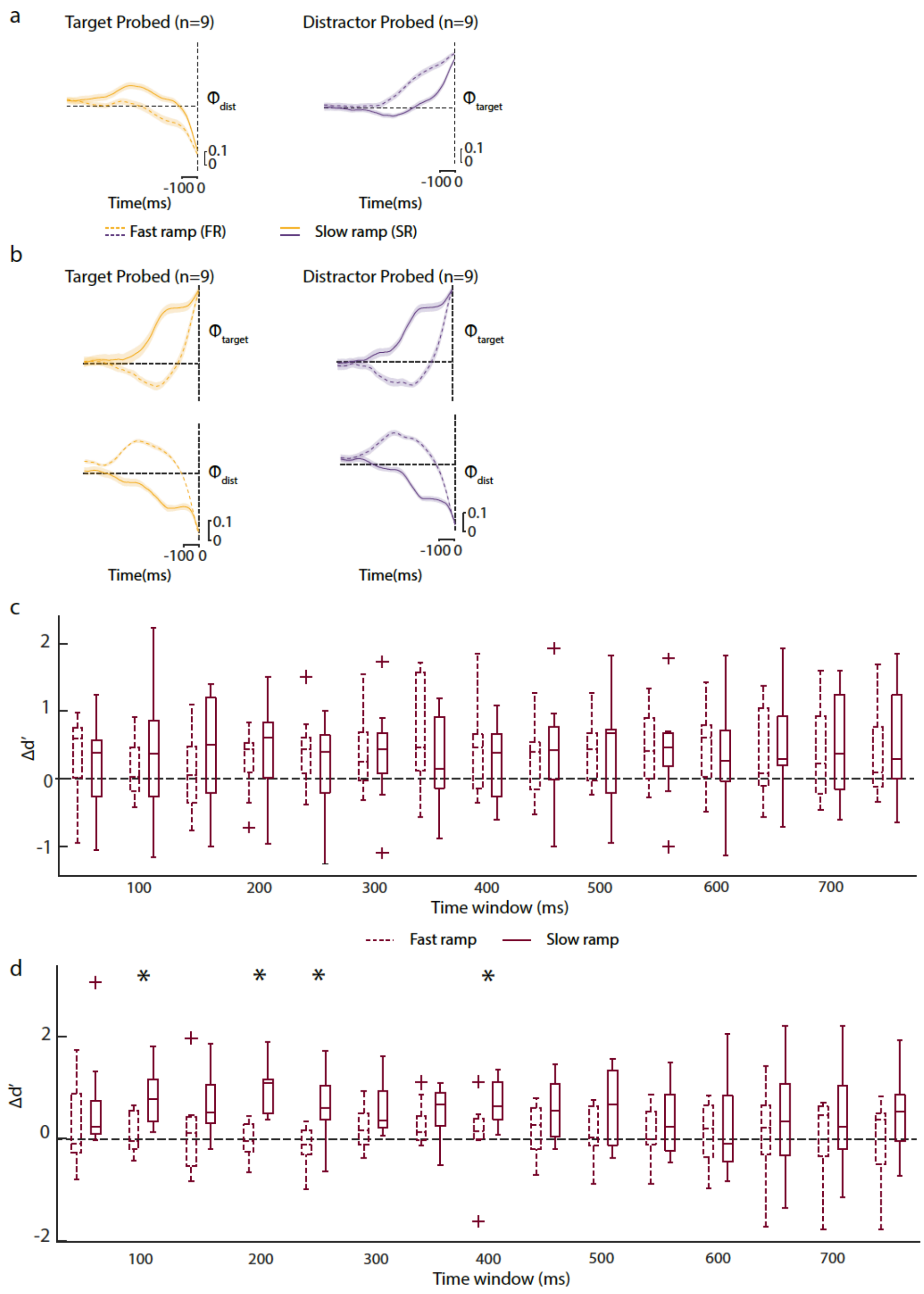

Supplementary Figure 5 Results auxiliary to Figure 5 (main text).

a. Same as in Fig. 5b but showing distractor and target Φ traces for target (Yellow) and distractor (Purple) probed trials respectively. Dashed and solid lines: fast ramping trials (n=9) and slow ramping trials (n=9), respectively. Trials pooled across subjects for Paradigm B. Shaded regions: s.e.m.

b. Same as in Fig. 5e but showing target (top) and distractor Φ (bottom) traces. Other conventions are the same as in panel Supplementary Figure 5a.

c. Same as in Fig. 5c (main text) but showing Δd' for all the time-windows that were used for computing the rate of change of Φ. Dashed and solid outline boxes: fast-ramping and

slow-ramping trials, respectively. Central mark: median, edges: 25th and 75th percentiles, the whiskers: most extreme points, +: outliers.

d. Same as in Fig. 5f but showing all the time-windows that were used for computing the rate of change of $\Delta\Phi$. Other conventions are the same as Supplementary Figure 5c. *$p<0.05$.

Supplementary Table 1. Flicker frequencies across participants.

Participant IDs	Flicker frequency
A1, A2, A3, A5, B2, B7, B8, B9, B10§	14 Hz (L), 18 Hz (R)
A4, B1, B3, B4, B5, B6, B11§	18 Hz (L), 14 Hz (R)
A6, A8, A11	13 Hz (E)
A7, A9, A10, A12*, A13*, A14*, A15*	15 Hz (E)

AX and BX denote participants ID-s for paradigms A and B, respectively. L: left hemifield; R: right hemifield; E-either hemifield. Flickering pedestals at 13 Hz or 15 Hz were presented on only one of the two hemifields. The other hemifield contained either a non-flickering (static) pedestal, or a pedestal flickering at 25 Hz (participants marked with an asterisk*). §B10 and B11 were the same participants as A4 and A5. Their data is not included in any analyses presented in the main text; only in Supplementary Figure 3c.

Supplementary Table 2. Summary of statistical analyses.

Figure Panel	Comparison	Type of Test	Statistic	Power
2A (inset)	SSVEP power index (Φ) between cued and uncued conditions	Non-parametric Wilcoxon signed-rank test	p-value	$p<0.01$
2B	Discrimination accuracy (d') between high-Φ and low-Φ trials	2-way ANOVA	p-value	$p<0.05$
	Choice criterion (c) between high-Φ and low-Φ trials			n.s.
	Reaction times between high-Φ and low-Φ trials			n.s.
2C	Discrimination accuracy (d') between high-Φ and low-Φ trials		p-value	n.s.
	Choice criterion (c) between high-Φ and low-Φ trials			n.s.
	Reaction times (RT) between high-Φ and low-Φ trials			n.s.
3B, insets	Differences between ΔΦ distributions	Kolmogorov-Smirnov test	p-value	$p<0.001$
3B	d' differences between ΔΦ distributions	4-way ANOVA	p-value	n.s.
4E	Discrimination accuracy (d') between target and distractor probed trials	Estimation statistics analog of the paired t-test	p-value	$p<0.01$
	Choice criterion (c) between target and distractor probed trials			$p<0.01$
	Reaction times (RT) between target and distractor probed trials			n.s.
5C	Changes between the Δd' value for fast-ramping versus slow-ramping trials based on Φ dynamics	Wilcoxon paired signed-rank test	p-value	n.s.
5F	Changes between the Δd' value for fast-ramping versus slow-ramping trials ΔΦ dynamics	Wilcoxon paired signed-rank test	p-value	$p<0.05$

Summary of the statistical analyses performed in the main text. The figure panel, the measures compared, the statistical test, corresponding statistic, and the power are tabulated. n.s = not significant.
