# Supplementary material for: Tracking momentary fluctuations in human attention with a cognitive brain-machine interface
Source: Commun Biol. 2022 Dec 8;5:1346. doi: 10.1038/s42003-022-04231-w (PMC9732358; doi:10.1038/s42003-022-04231-w)
Supplement: Supplementary file 3 — Description of Additional Supplementary Files [file 42003_2022_4231_MOESM3_ESM.pdf]

## Description of Additional Supplementary Files

**File name:** Supplementary Data 1
**Description:** Statistical detail information for figures

Fig 2a: Mean Φ for cued and uncued trials in the interval -500 ms-0ms before stimulus onset for right and left SSVEPs (n=15).
Fig 2bi: Discrimination accuracy (d') for high-Φ and low-Φ trials for the target-side triggered trials, for right and left SSVEPs (n=15).
Fig 2bii: Same as Fig 2bi, but for choice criterion (c).
Fig 2biii: Same as Fig 2bi, but for reaction times.
Fig 2ci-iii: Same as Fig 2bi-iii, but for distractor-side triggered trials (n=10).

Fig 3bi: d' values for Extreme (Ext) +ΔΦ and moderate (Mod) ΔΦ for high-Φ, target side triggered trials (n=5).
Fig 3bii: d' values for Extreme (Ext) -ΔΦ and moderate (Mod) ΔΦ for low-Φ, target side triggered trials (n=5).
Fig 3biii: d' values for Extreme (Ext) -ΔΦ and moderate (Mod) ΔΦ for high-Φ, distractor side triggered trials (n=4).
Fig 3biv: d' values for Extreme (Ext) +ΔΦ and moderate (Mod) ΔΦ for low-Φ, distractor side triggered trials (n=4).

Fig 4ei: d' for target-side probed and distractor-side probed trials for Paradigm B (n=9).
Fig 4eii: Same as Fig 4ei, but for criterion (c) (n=9).
Fig 4eiii: Same as Fig 4ei, but for reaction times (n=9).

Fig 5c: Δd' for fast ramping (FR) and slow ramping (SR) subsets, for Φ dynamics (n=9).
Fig 5d: Δd'(SR – FR) for time windows from 50 to 750 ms, in steps of 50 ms, for Φ dynamics (n=9).
Fig 5f-g: Same as Fig 5c-d but for ΔΦ dynamics (n=9).
