# Supplementary material for: Tracking momentary fluctuations in human attention with a cognitive brain-machine interface
Source: Commun Biol. 2022 Dec 8;5:1346. doi: 10.1038/s42003-022-04231-w (PMC9732358; doi:10.1038/s42003-022-04231-w)
Supplement: Supplementary file 5 — Reporting Summary [file 42003_2022_4231_MOESM5_ESM.pdf]

nature portfolio

Corresponding author(s): Devarajan Sridharan

Last updated by author(s): Oct 26, 2022

Reporting Summary

Nature Portfolio wishes to improve the reproducibility of the work that we publish. This form provides structure for consistency and transparency in reporting. For further information on Nature Portfolio policies, see our Editorial Policies and the Editorial Policy Checklist.

Statistics

For all statistical analyses, confirm that the following items are present in the figure legend, table legend, main text, or Methods section.

n/a	Confirmed	
☐	☒	The exact sample size (*n*) for each experimental group/condition, given as a discrete number and unit of measurement
☐	☒	A statement on whether measurements were taken from distinct samples or whether the same sample was measured repeatedly
☐	☒	The statistical test(s) used AND whether they are one- or two-sided<br>*Only common tests should be described solely by name; describe more complex techniques in the Methods section.*
☒	☐	A description of all covariates tested
☐	☒	A description of any assumptions or corrections, such as tests of normality and adjustment for multiple comparisons
☐	☒	A full description of the statistical parameters including central tendency (e.g. means) or other basic estimates (e.g. regression coefficient) AND variation (e.g. standard deviation) or associated estimates of uncertainty (e.g. confidence intervals)
☐	☒	For null hypothesis testing, the test statistic (e.g. *F*, *t*, *r*) with confidence intervals, effect sizes, degrees of freedom and *P* value noted<br>*Give P values as exact values whenever suitable.*
☐	☒	For Bayesian analysis, information on the choice of priors and Markov chain Monte Carlo settings
☒	☐	For hierarchical and complex designs, identification of the appropriate level for tests and full reporting of outcomes
☐	☒	Estimates of effect sizes (e.g. Cohen's *d*, Pearson's *r*), indicating how they were calculated

Our web collection on statistics for biologists contains articles on many of the points above.

Software and code

Policy information about availability of computer code

Data collection	MATLAB 2015b, Psychtoolbox 3, Fieldtrip 20170817, Chronux 2.12v03, Biosemi Actiview 8.11, Labstreaminglayer, Openvibe 1.2.2, GPFA v0203, Noisetoolbox version 21-Mar-2016.
Data analysis	MATLAB 2017a and 2019a, Fieldtrip 20170817, Chronux 2.12v03, EEGLab 14.0.0b, kakearney/boundedline-pkg, GPFA v0203, Noisetoolbox version 21-Mar-2016, robustcorr toolbox 2012.

For manuscripts utilizing custom algorithms or software that are central to the research but not yet described in published literature, software must be made available to editors and reviewers. We strongly encourage code deposition in a community repository (e.g. GitHub). See the Nature Portfolio guidelines for submitting code & software for further information.

Data

Policy information about availability of data

All manuscripts must include a data availability statement. This statement should provide the following information, where applicable:

- Accession codes, unique identifiers, or web links for publicly available datasets
- A description of any restrictions on data availability
- For clinical datasets or third party data, please ensure that the statement adheres to our policy

All data and code necessary for reproducing all figures in the paper have been deposited into an opensource online repository (FigShare; https://dx.doi.org/10.6084/m9.figshare.13720546) for ready inspection and replication of the results.

Human research participants

Policy information about studies involving human research participants and Sex and Gender in Research.

Reporting on sex and gender	Due to low sample size, no sex and gender based analyses were performed.
Population characteristics	Twenty-four subjects (9 females; age range: 20-28 years; median age: 23 years) with no known history of neurological disorders and with normal or corrected-to-normal vision participated in the experiment. Most of the subjects were university undergraduate or graduate students.
Recruitment	Participants were recruited by word of mouth and advertisement posters in the university.
Ethics oversight	Institute Human Ethics Committee at the Indian Institute of Science, Bangalore.

Note that full information on the approval of the study protocol must also be provided in the manuscript.

Field-specific reporting

Please select the one below that is the best fit for your research. If you are not sure, read the appropriate sections before making your selection.

☒ Life sciences ☐ Behavioural & social sciences ☐ Ecological, evolutionary & environmental sciences

For a reference copy of the document with all sections, see nature.com/documents/nr-reporting-summary-flat.pdf

Life sciences study design

All studies must disclose on these points even when the disclosure is negative.

Sample size	N=24 unique participants. N=15 participants for Paradigm A, N=11 participants for Paradigm B, and N=2 participants overlapping across paradigms. The sample for each paradigm was determined by power analysis. Details are provided in SI Methods section on "Sample size estimation".
Data exclusions	Data from the 2 subjects in paradigm B that were also in paradigms A were excluded from analyses in the main text. Results including both of those subjects also is presented in the SI.
Replication	We conducted two sets of experiments; key results from paradigm A were replicated in paradigm B.
Randomization	The study involved a within subject experimental design. All conditions varied randomly over trials.
Blinding	The study involved a within subject experimental design. All conditions (high-phi vs low-phi, target vs distractor) were blinded for both the experimenter and the subject.

Reporting for specific materials, systems and methods

We require information from authors about some types of materials, experimental systems and methods used in many studies. Here, indicate whether each material, system or method listed is relevant to your study. If you are not sure if a list item applies to your research, read the appropriate section before selecting a response.

Materials & experimental systems

n/a	Involved in the study	
☒	☐	Antibodies
☒	☐	Eukaryotic cell lines
☒	☐	Palaeontology and archaeology
☒	☐	Animals and other organisms
☒	☐	Clinical data
☒	☐	Dual use research of concern

Methods

n/a	Involved in the study	
☒	☐	ChIP-seq
☒	☐	Flow cytometry
☒	☐	MRI-based neuroimaging
